# seekCRIT: Detecting and characterizing differentially expressed circular RNAs using high-throughput sequencing data

**DOI:** 10.1371/journal.pcbi.1008338

**Published:** 2020-10-20

**Authors:** Mohamed Chaabane, Kalina Andreeva, Jae Yeon Hwang, Tae Lim Kook, Juw Won Park, Nigel G. F. Cooper

**Affiliations:** 1 Department of Computer Science and Engineering, University of Louisville, Louisville, Kentucky, United States of America; 2 Department of Anatomical Sciences and Neurobiology, University of Louisville, Louisville, Kentucky, United States of America; 3 KBRIN Bioinformatics Core, University of Louisville, Louisville, Kentucky, United States of America; Johns Hopkins University, UNITED STATES

## Abstract

Over the past two decades, researchers have discovered a special form of alternative splicing that produces a circular form of RNA. Although these circular RNAs (circRNAs) have garnered considerable attention in the scientific community for their biogenesis and functions, the focus of current studies has been on the tissue-specific circRNAs that exist only in one tissue but not in other tissues or on the disease-specific circRNAs that exist in certain disease conditions, such as cancer, but not under normal conditions. This approach was conducted in the relative absence of methods that analyze a group of common circRNAs that exist in both conditions, but are more abundant in one condition relative to another (differentially expressed). Studies of differentially expressed circRNAs (DECs) between two conditions would serve as a significant first step in filling this void. Here, we introduce a novel computational tool, seekCRIT (seek for differentially expressed CircRNAs In Transcriptome), that identifies the DECs between two conditions from high-throughput sequencing data. Using rat retina RNA-seq data from ischemic and normal conditions, we show that over 74% of identifiable circRNAs are expressed in both conditions and over 40 circRNAs are differentially expressed between two conditions. We also obtain a high qPCR validation rate of 90% for DECs with a FDR of < 5%. Our results demonstrate that seekCRIT is a novel and efficient approach to detect DECs using rRNA depleted RNA-seq data. seekCRIT is freely downloadable at https://github.com/UofLBioinformatics/seekCRIT. The source code is licensed under the MIT License. seekCRIT is developed and tested on Linux CentOS-7.

## Author summary

The focus of circRNA studies has been on condition-specific circRNAs, however, there are situations in which circRNAs exist in both conditions with different abundance. Here, we introduce a new and robust analytic software, seekCRIT (seek for differentially expressed CircRNAs In Transcriptome), that identifies the differentially expressed circRNAs (DECs) between two conditions from high-throughput sequencing data. seekCRIT provides a straightforward normalized quantification of circRNAs and statistical measures by adapting a junction-count-based estimation approach. Using publicly available ribosomal RNA depleted RNA-seq data and our own rat retina RNA-seq data, we show that seekCRIT can efficiently detect circRNAs and identify DECs. We also obtain a high qPCR validation rate of 90% for DECs with a FDR of < 5%. Our results demonstrate that seekCRIT is a novel and efficient software to detect DECs using rRNA depleted RNA-seq data.

This is a *PLOS Computational Biology* Software paper.

## Introduction

**Fig 1:**
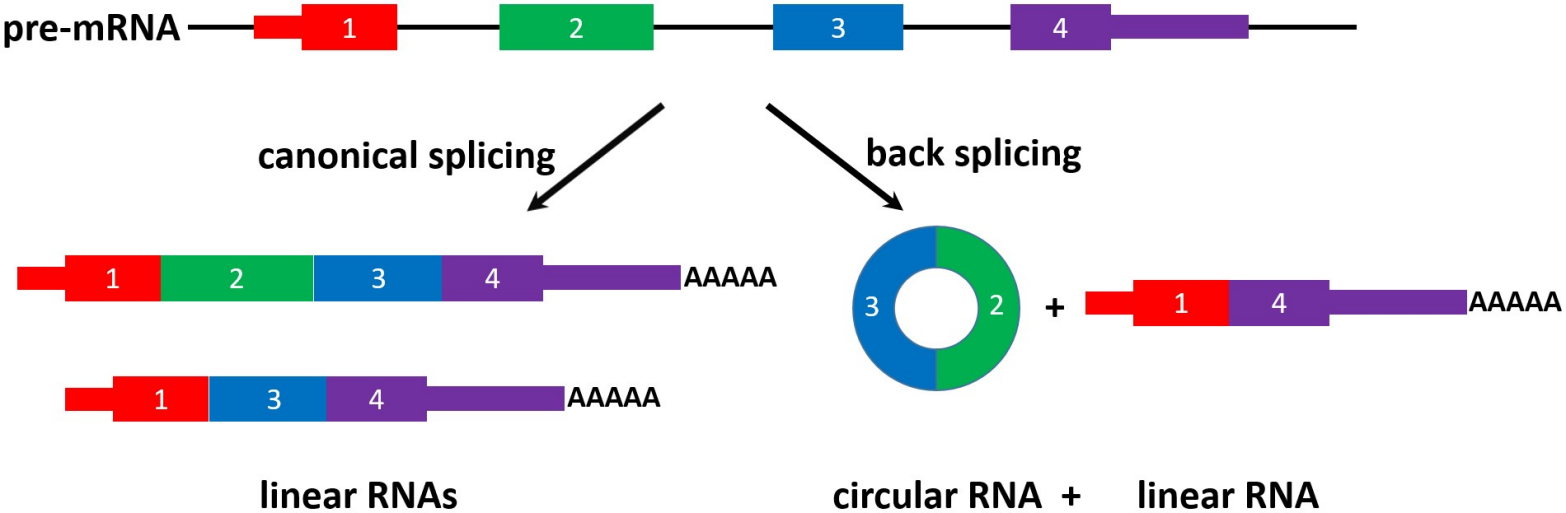
Illustration of linear RNA and circRNA formation.

Alternative splicing (AS) refers to the production of multiple mRNA isoforms from a single gene due to alternative selection of exons or splice sites during pre-mRNA splicing. It is a primary mechanism of gene regulation in higher eukaryotes and significantly expands the functional complexity of eukaryotic organisms [1]. While canonical alternative splicing produces a linear form of RNA by joining an upstream donor site (5’ splice site) with a downstream acceptor site (3’ splice site) within a single intron, a special form of alternative splicing (back-splicing) produces a circular form of RNA by ligating a downstream donor site of the flanking downstream intron with an upstream acceptor site of a second upstream intron (Fig 1) [2].

Although these circRNAs have garnered considerable attention in the scientific community due to their major role as a microRNA (miRNA) activity modulator [3,4] and their association with various diseases including cancer [5–10], the focus of these studies has been on condition-specific circRNAs where the circRNAs are present in one condition and entirely absent in the other. For example, the circRNAs may exist only in one tissue but not in other tissues (tissue-specific) or exist in certain disease conditions, such as cancer, but not under normal conditions (disease-specific). In addition to these condition-specific circRNAs, there are situations in which circRNAs exist in both conditions (common circRNA) but are more abundant in one condition than the other condition (differentially expressed). These common, differentially expressed circRNAs (DECs) are expected to play an important role in the mRNA regulatory network by proportionally binding to miRNAs according to its abundance. Moreover, the distinction between condition-specific and common circRNAs may partly disappear as detection sensitivity increases due to the utilization of higher RNA-seq read depths and other sensitivity-enhancement approaches. In our experience, over 75% of the detected circRNA is common in both. Consequently, the development of a computational tool for identifying and characterizing DECs from both condition-specific circRNAs and common circRNAs is critical to helping researchers understand the role of circRNAs in regulation associated with various diseases.

Several computational tools for identification of circRNAs from sequencing data have been developed, including MapSplice[11], DCC [12], NCLscan [13], CIRI [14], find_circ [15], UROBORUS[16], KNIFE [17], circRNA_finder [18], CIRCexplorer [19], and CIRCexplorer2 [20]. These tools examine chimeric reads (i.e., reads that map to two distinct portions of the genome) to identify circular junction reads that span a back-spliced junction. Although these tools have been widely used in many studies for the identification of circRNAs[15,21–28], and list over half a million circRNAs in online databases [29–32], these tools only address the circRNA detection issue. Also, current computational approaches for the quantification of circRNAs, whether based on the number of circular junction reads that span the back splicing junctions [17,26] or on a model that considers isoforms and reads from a given isoform [33], have serious limitations for systematic detection of DECs because the current tools focus on quantifying the expression levels of circRNAs within the sample not across the samples. Other computational tools for differentially expressed gene (DEG) studies [34–37] have been developed to quantify gene expression and detect DEGs from RNA-seq. However, these tools are not applicable to DEC detection because they cannot account for the back-spliced junctions specific to circRNAs. Therefore, there is a need for new and robust analytic tools to systematically quantify circRNAs with respect to linear forms and identify DECs using RNA-seq data.

Here, we introduce seekCRIT (seek for differentially expressed CircRNAs In Transcriptome), a novel computational tool for the identification of DECs between two biological conditions using ribosomal RNA (rRNA) depleted RNA-seq data. seekCRIT has many distinct features over existing computational approaches. First, seekCRIT offers a simple automated platform for the detection of DECs between two biological conditions. seekCRIT can identify a set of DECs using RNA-seq data (fastq files) from each condition, genome (fasta), and transcriptome (gtf or refSeq). This simple input requirement allows researchers to identify DECs with no complicated steps. Second, seekCRIT can work with replicates. This feature is important because it has become customary to generate hundreds of millions of RNA-seq data with replicates due to a dramatic drop in high-throughput sequencing cost. Finally, and most importantly, seekCRIT provides a straightforward normalized quantification of circRNAs and statistical measures by adapting a junction-count-based estimation approach [38–41]. This approach naturally offers the normalized quantification because the circular junction and the linear junction have the same mappable lengths. To evaluate the performance of seekCRIT, we analyzed three sets of deep rRNA depleted RNA-seq data from rat brain, rat retina, and human umbilical vein. Based on the seekCRIT results from rat retina data, we randomly selected 15 circRNAs to cover a wide range of FDR values for qPCR validation. For circRNAs with a seekCRIT FDR of <5%, we obtained a high validation rate of 90%, demonstrating that seekCRIT can reliably detect DECs.

## Design and implementation

### Overview of seekCRIT

**Fig 2:**
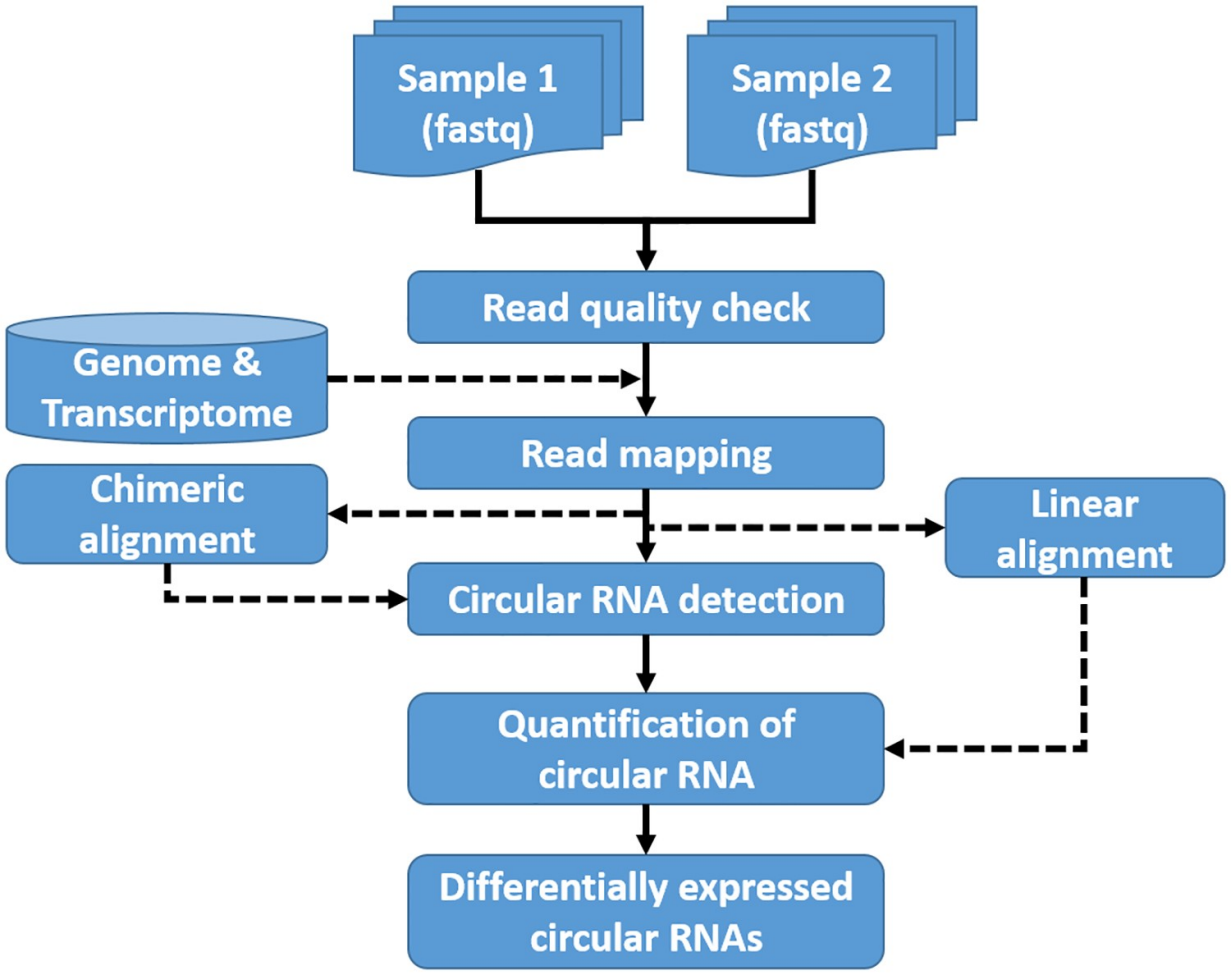
Overall workflow of seekCRIT. seekCRIT takes raw sequence files in fastq format to detect differentially expressed circRNAs.

seekCRIT is implemented as an automated pipeline for the identification of DECs between two biological conditions. Fig 2 illustrates the overall workflow of seekCRIT. seekCRIT requires the following inputs:

- *High-throughput sequencing data (fastq)*: seekCRIT pipeline begins with raw RNA-seq data from the rRNA depleted library preparation.
- *Genome sequence (fasta)*: To facilitate the use of seekCRIT, genomic sequences for various species are provided.
- *Transcriptome annotation*: This annotation file has all information for genes, transcripts, exons, and introns for the given species. Back-splicing and canonical linear splicing are determined based on this annotation. The annotation file should be in the commonly used GTF (Gene Transfer Format) format or refSeq format. Both GTF files and refSeq files for various species are provided.
- Optional parameters to specify minimum number of circular junction count (CJC) for high-confidence circRNAs and to specify the number of threads to use for faster running time: To increase the sensitivity of circRNA detection, we allow users to require a minimum number of CJC. Users can also specify the number of threads they want to use for an improved running time.

Given proper inputs, seekCRIT performs a series of automated processes to uncover the DECs. In short, it enables users to check the quality of raw sequences, map reads to genome and transcriptome, find chimeric and linear alignments, detect circRNAs, estimate expression levels per circRNA per sample, compute statistical measures (p-value and FDR) per circRNA, and identify DECs at a user defined FDR cut-off, typically 5%.

### Estimating circRNA expression levels or Percent-Backspliced-In (PBI)

$${PBI}_{sample}=\frac{{CJC}_{sample}}{\left({CJC}_{sample}+\frac{{LJC}_{sample}}{2}\right)}$$ (1)

Eq 1 PBI calculation.

**Fig 3:**
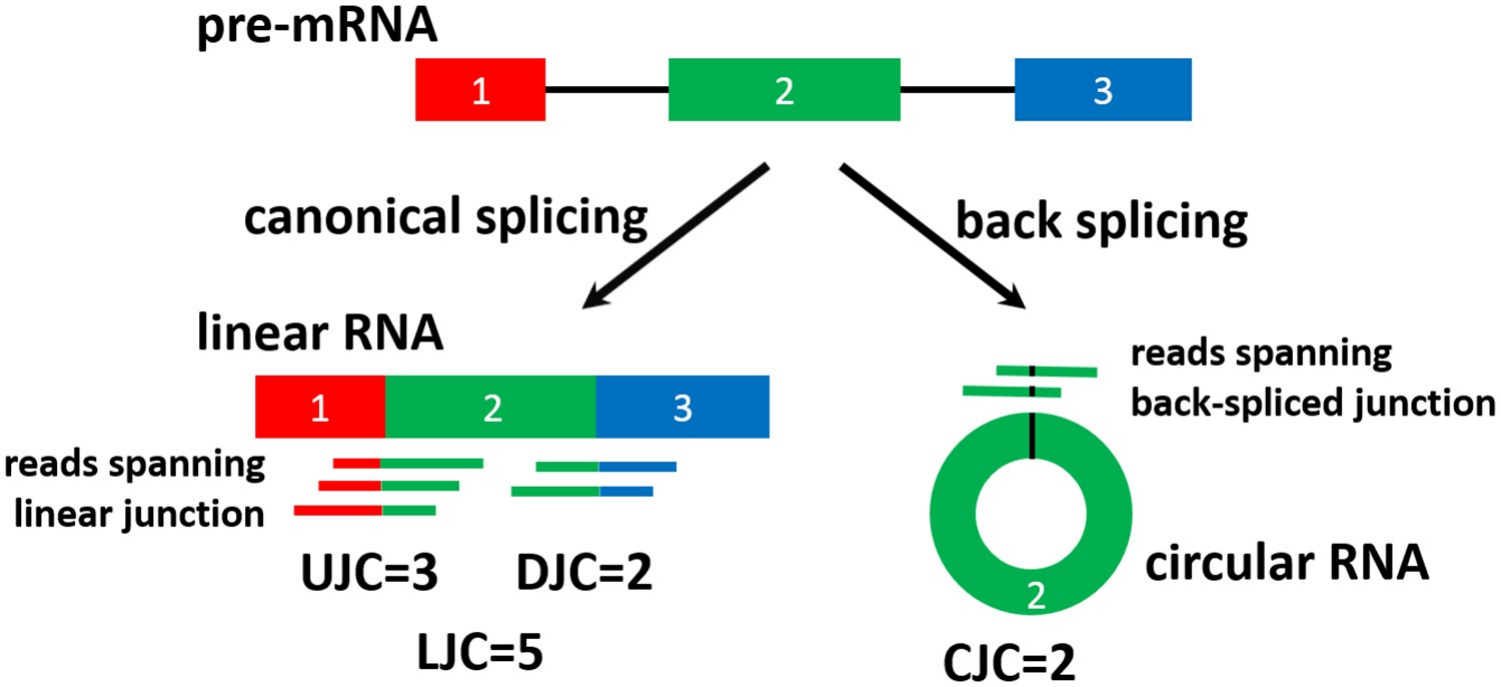
Different types of junction counts. UJC: upstream junction count, DJC: downstream junction count, LJC: linear junction count (UJC+DJC), CJC: circular junction count.

Similar to the exon inclusion level (PSI or ψ) estimate for alternatively spliced cassette exons [38–41], we define the expression level (Percent-Backspliced-In, or PBI) of a circRNA as the percentage of circRNA transcripts among all such circRNA transcripts plus linear transcripts that splice from its upstream flanking exon directly into its first exon or from its last exon directly into its downstream flanking exon. In an RNA-seq study, for circRNA in a given sample we count the number of RNA-seq reads uniquely mapped to its circular, upstream junction, or downstream junctions (Fig 3). The upstream junction count (UJC in Fig 3) and the downstream junction count (DJC in Fig 3) reflect the abundance of the linear RNA isoform, while the circular junction count (CJC in Fig 3) reflects the abundance of the circRNA isoform. Let *CJC* and *LJC* (Linear Junction Count) represent the counts of circRNA and linear RNA isoforms respectively. Assuming that the read counts follow a binomial distribution, the maximum likelihood estimate (MLE) of the expression level (PBI) of a circRNA in a given sample can be calculated as Eq 1.

### circRNA detection in each sample

In the circRNA detection process, seekCRIT first maps reads to genome and transcriptome using the STAR aligner [42]. Since the key idea of circRNA detection is to detect reads that span back-spliced junction, seekCRIT examines chimeric alignments (possibly from gene fusion events or circularization) to detect circular RNAs following the algorithm described in [43]. The algorithm examines all chimeric alignments and unmapped reads to find cases where a read has two segments and these segments aligned separately within a gene but in reverse order. For example, the first 30bp of a 100bp read map to the 3’ end of an exon, while the remaining 70bp map to the 5’ end of an exon. To ensure the circRNA detection from seekCRIT is equivalent or comparable to other tools, we detected circRNAs from somata layers of CA1 hippocampal region using CIRI2, CIRCExplorer2, find_circ, and seekCRIT. As shown in Fig B in S1 Text, majority of detected circRNAs overlap among the tools.

### Upstream junction count (UJC) and downstream junction count (DJC)

UJC and DJC are required to compute PBI. seekCRIT examines mapped reads in the bam files to detect upstream junction count and downstream junction count associated with the detected circular RNAs. For each circular RNA, seekCRIT examines the bam file and compiles number of reads that span upstream junction (for UJC) and downstream junction (for DJC) of the given circular RNA.

### p-value, FDR, and DECs

seekCRIT uses Fisher’s exact test to calculate the p-value. For each circRNA, the CJC and LJC for each sample are computed from chimeric alignments and linear alignments, respectively. From these values, seekCRIT constructs the 2x2 table (Table 1) to compute the Fisher’s exact test p-value per circRNA. After obtaining the p-values of all circRNAs, the Benjamini–Hochberg method [44] is applied to obtain the corrected p-value or false discovery rate (FDR).

**Table 1:** 2x2 table used for Fisher’s exact test.

2*CJC_sample_1_	LJC_sample_1_
2*CJC_sample_2_	LJC_sample_2_

To detect DECs between two samples, for each circRNA, we define PBI_1_ and PBI_2_ as its expression levels in sample 1 and sample 2, respectively. We then define the circRNA expression level difference, deltaPBI (or ΔPBI), as PBI_1_—PBI_2_. seekCRIT then examines all circRNAs and identifies DECs that meet |ΔPBI| ≥ 5% and FDR < 5%.

### RNA-seq data sets

**Fig 4:**
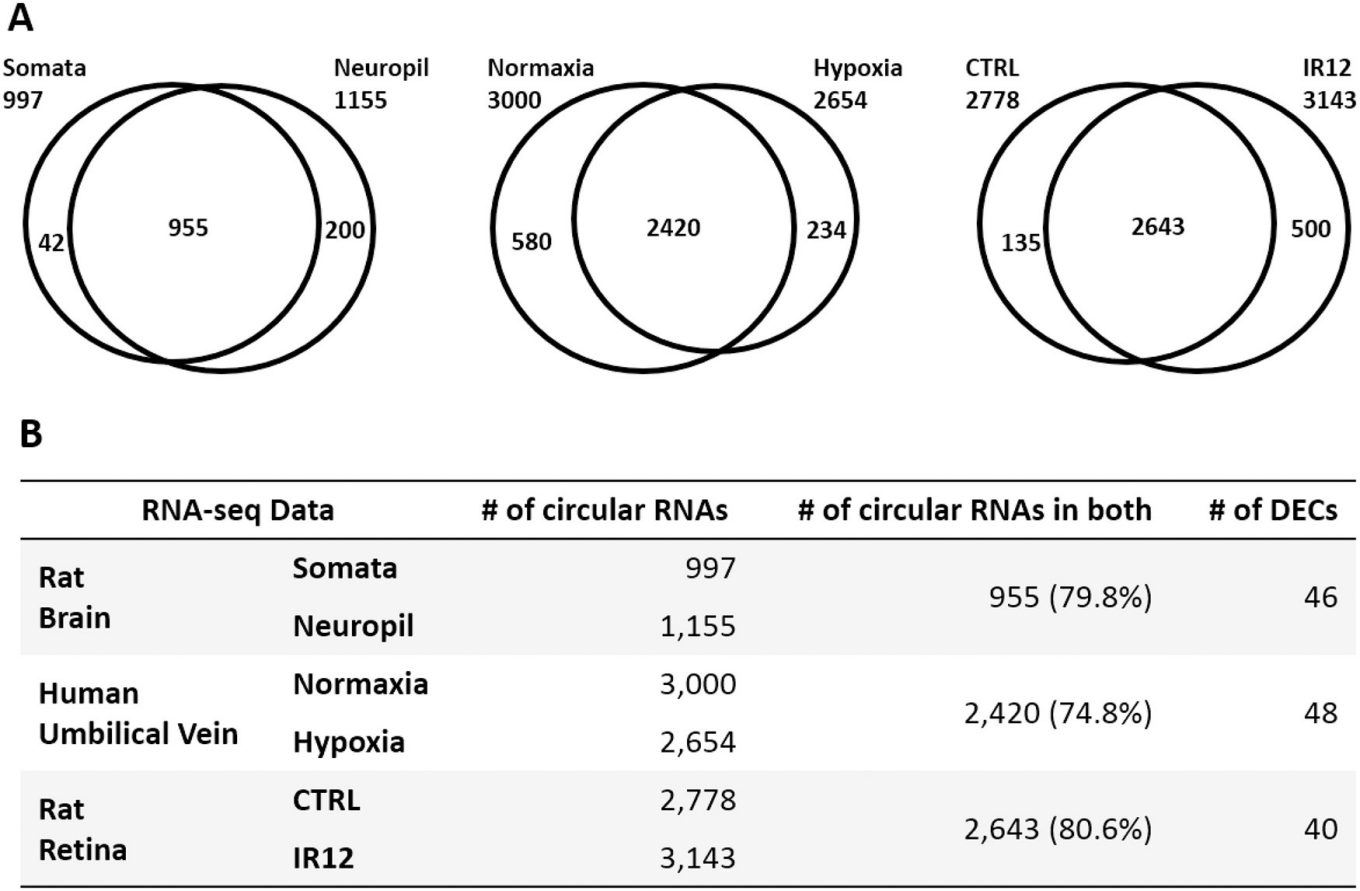
Composition of circRNAs. All three datasets showed that majority of circRNAs are expressed in both biological samples compared. DECs meet both |ΔPBI| > 5% and FDR < 5%.

To demonstrate the applicability of seekCRIT, we analyzed three rRNA depleted RNA-seq datasets (Fig 4): two public datasets from the NCBI Gene Expression Omnibus (GEO) and one in-house data set. First, to demonstrate that seekCRIT can work with/without replicates, we analyzed a couple of NCBI GEO datasets. One of the GEO datasets is from rat brain tissues (somata and neuropil layers of CA1 hippocampal region, no replicates, GSE61991). This 151 bp single-end dataset was generated from the HiSeq 2500. Somata sample (SRR1772434) has 38.9 million reads and neuropil sample (SRR1772433) has 39.7 million reads. This dataset was selected because circRNA is known to be highly abundant in neural tissues [45]. The other GEO data is from human umbilical vein endothelial cells (12 hour normal and hypoxia conditions, two replicates, GSE107029). This paired-end dataset (94 bp in read 1, 100 bp in read 2) was generated from the HiSeq 2500 as well. Since it is not common to have different read length for read 1 and read 2, we examined GEO’s metadata, fastq-dump commands used for downloading, and raw fastq files generated from SRA to confirm that read 1 and read 2 indeed have different read lengths. Normaxia samples (SRR6300671 and SRR6300672) have 42.5 million pairs on average and hypoxia sample (SRR6300667 and SRR6300668) have 41.5 million pairs on average. Each sample has two replicates and it was confirmed that circRNAs are abundant in endothelial cells [46]. Next, rRNA depleted RNA-seq is generated from rat retina for the validation of DECs identified from seekCRIT. We used two retinas extracted from Long-Evans rats subjected to ischemia-reperfusion (IR) injury as previously described [47–49]. In this study we sequenced two retinal samples: a sample subjected to IR injury and collected 12 hours after the reperfusion (IR12) and a control sample (CTRL) subjected to a sham-procedure. The resulting RNA-seq dataset consisted of 206.7 million single-end reads, including 113.9 million reads for the rRNA depleted IR12h sample, and 92.8 million reads for the rRNA depleted control sample. Due to the low read qualities near 3’ end of our RNA-seq reads, we decided to use the first 100 bp of each read in this study. The RNA-seq data has been deposited into the GEO under the accession number GSE114896.

### Validation of identified DECs via qPCR

To assess the overall accuracy of our PBI estimates and DECs detection, we randomly selected 15 circRNAs covering three categories; five from upregulated: ΔPBI < -5%, five from downregulated: ΔPBI > 5%, and five from unchanged: |ΔPBI| < 5%. 10 circRNAs in upregulated and downregulated categories meet FDR < 5% as well.

### Simulation study

To systematically assess the accuracy of seekCRIT and its statistical measure, we generated simulated data and used gold-standard DEC set obtained from the simulated data to calculate the true positive rate (i.e., sensitivity) of seekCRIT under various read lengths and read depths. Our gold-standard DEC set has 1055 DECs and it allowed us to investigate factors that could influence the sensitivity of seekCRIT. To assess the importance of sequencing depth and read length on detection of DEC, we randomly generated RNA-Seq data from human (hg38) and estimated the sensitivity under various levels of sequencing depth and read length. Each simulated RNA-seq consists of 5% of reads from circular RNA, 91.5% of reads from linear mRNA, 2% of reads from intron, 1% of reads from intergenic region, and 0.5% of reads from sequencing error. We performed 10 independent random samplings at each sequencing depth (from 10 million to 120 million reads) and at each read length (from 50 bp to 150 bp). As expected, we observed low sensitivity at lower levels of sequencing depth, especially when the number of reads per sample was below 40M, and at lower levels of read length (Fig F in S1 Text). Our simulation study suggests that running seekCRIT with 20-40M reads per sample are barely scratching the surface of circular RNA variations. Our simulation study also provides that seekCRIT and its statistical model perform well with over 50M reads per sample and with read length > 75 bp.

### Ethics statement

Long Evan’s female rats (weighing 250–300 g) were used in this study. The animals were housed on a 12-hour light-dark cycle with food and water ad libitum. The treatment and care of all animals were approved by the University of Louisville Institutional Animal Care and Use Committee (IACUC 12062) and were performed in accordance with the Association for Research in Vision and Ophthalmology Statement for the Use and Care of Animals in Ophthalmic and Vision Research.

## Results

### High abundance of common circRNAs

We examined three RNA-seq datasets and found that majority (at least 74% in our datasets) of the detected circRNAs are commonly expressed in both biological samples (Fig 4). Thus, it strongly supports the critical need for a computational tool to examine and detect DECs between two biological samples using high-throughput sequencing data.

### seekCRIT analysis of publicly available rRNA depleted RNA-seq data

**Fig 5:**
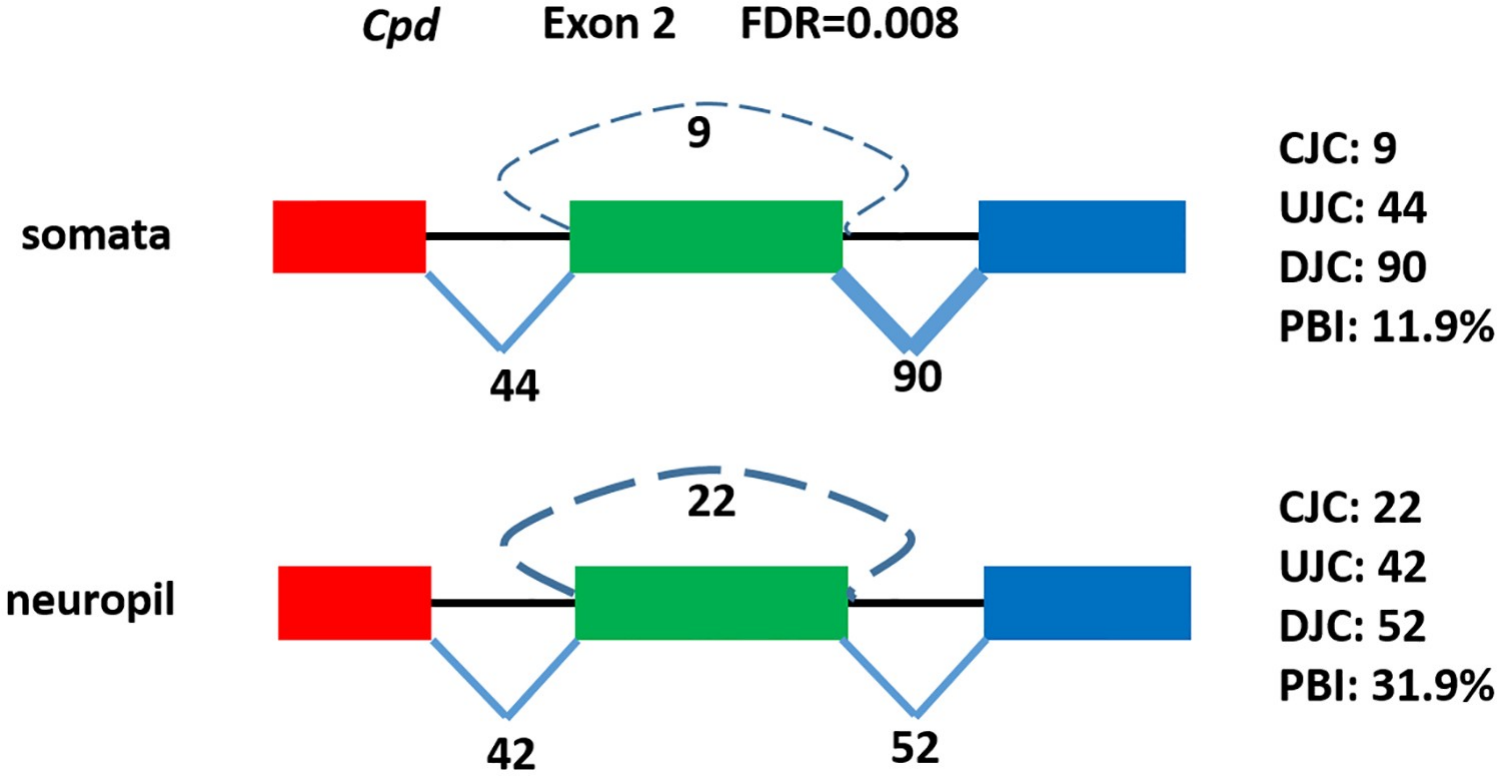
An example of differentially expressed circRNA (green exon). Its ratio of circRNA to linear RNA (PBI) is higher in neuropil sample than in somata sample.

To show that seekCRIT can work with/without replicates, two publicly available datasets from the tissues that were known to have high abundance of circRNAs were obtained from the NCBI GEO. The first data set contains somata and neuropil samples from rat brain. Both samples have about 40 million 151bp single-end reads which are deep enough to conduct splicing studies for highly or moderately expressed genes in eukaryotes [50–52]. For each of 1,197 circRNAs identified with more than or equal to 3 circular junction reads in at least one sample, the ratio of circular form to the sum of circular and linear forms (PBI) is computed as described in Eq 1 for each sample. In addition to the PBI calculation, a 2x2 table (see Table 1) is used to compute the Fisher’s exact p-value and Benjamini-Hochberg FDR per circRNA. At FDR 5%, seekCRIT identified 46 DECs that showed at least 5% differential expression between somata and neuropil, or |ΔPBI|>5% (see **Table in** S1 Table). An example of DEC in *Cpd* is shown in Fig 5. All of the 46 DECs identified from seekCRIT are highly expressed circRNAs as they have at least 5 back-spliced junction reads (i.e., CJC> = 5) similar to Yang’s previous study [19]. Since circRNAs are found to be more enriched in neuropil than in somata [45], it was encouraging to see that all 46 DECs have a higher expression level in the neuropil than in the somata.

A second publicly available dataset contains human umbilical vein samples under a normal oxygen level (20%, normaxia) and a low oxygen level (0.2%, hypoxia). Each sample has two replicates with ~42 million paired-end reads (94 bp read 1, 100 bp read 2) per replicate. seekCRIT detected over 3,000 circRNAs and for each circRNA, seekCRIT computed PBI per sample by combining the junction counts from each replicate. At FDR 5% and |ΔPBI|>5%, seekCRIT identified 48 DECs (see **Table in** S1 Table), and of these, 46 are highly expressed circRNAs (at least one sample has 5 or more CJC).

### seekCRIT analysis of rat retina data

To evaluate the overall accuracy of seekCRIT, we used seekCRIT to detect DECs using our rRNA depleted RNA-seq data generated from rat retina samples. Our RNA-seq data contains a control sample (CTRL) and an ischemic injury sample collected 12 hours after the injury (IR12). Each sample has ~100 million 150 bp single-end reads and we truncated the last 50 bps due to their low read quality. seekCRIT detected 3,278 circRNAs and 40 of these were DECs at FDR 5% and |ΔPBI|>5% (see **Table in** S1 Table).

### RT-qPCR validation of seekCRIT results

Validation of deregulated circRNAs RT-qPCR assay was performed to test the computational predictions of altered circRNA species in response to the retinal ischemic insult. The relative accumulation of 15 putative DECs with different size and abundance was experimentally validated. Fold changes were calculated to determine the level of expression of the circular junctions and their surrounding exon-exon linear junctions. The fold changes were then used to compute and compare the usage of the circRNA junctions in injured and sham control retinas 12h after ischemia-reperfusion injury (see **validation data in** S1 Text for more details). In 9 out of 10 validated cases (90% validation rate, 5 upregulated and 5 downregulated in IR12) PBIs calculated by seekCRIT detected the same directional change as the one determined by the RT-qPCR (**Table B in** S1 Text). Pearson’s correlation between the trends determined by seekCRIT and RT-qPCR indicated agreement (*r* = 0.71) (see **Fig C in** S1 Text).

## Discussion

All three datasets showed that a majority of the detected circRNAs exist in both biological conditions. It clearly indicates that the development of a computational tool for identifying DECs from both condition-specific circRNAs and common circRNAs will increase the number of identifiable DECs significantly. In turn, it will have a substantial impact on helping researchers understand the role circRNAs play in regulation.

Our RT-qPCR validation showed 90% validation rate for the DECs tested and it also showed high correlation between the seekCRIT PBI estimate and the RT-qPCR PBI estimate. It supports that seekCRIT can reliably detect DECs from RNA-seq data. We also noticed that two of the 5 control circRNAs (for which seekCRIT detected no change) showed more than 5% changes in the RT-qPCR results. We believe false negatives arise for circRNAs with low expression due to a systematic bias in RNA-seq. This bias has been addressed in other high-throughput sequencing studies as well [53].

Some previous research has found that one gene could generate multiple circular RNAs [20]. To verify that seekCRIT can detect such phenomenon we examined our results from rat brain tissue data (somata and neuropil layers of CA1 hippocampal region, no replicates, GSE61991) and found over 1000 cases. Two example cases from Galc (5 circular RNAs) and Ptbp2 (2 circular RNAs) genes are shown in **Fig E. in** S1 Text.

## Availability and future directions

seekCRIT is freely downloadable at https://github.com/UofLBioinformatics/seekCRIT. S1 File contains source code, documentation, instructions, and test data as well. The use of seekCRIT in DEC detection in the induced ischemic injury in rat retina along with the validation of identified DECs was illustrated here but seekCRIT can be applied to any rRNA depleted RNA-Seq data from two biological conditions. The regulatory roles of circRNA are important, but they are not well understood. In alternative splicing studies, it has been shown that RNA binding proteins (RBPs) play regulatory roles in alternative splicing, and several computational tools for RBP motif enrichment analysis have been developed [54]. Since circRNAs are produced from a special form of splicing, systematically generating RNA maps for examining the enrichment of RBP motifs and splicing factor motifs on or near circRNAs would have significant scientific value for better understanding the regulatory landscape.

## Supporting information

S1 Text Supplementary text, figures, and table. The details of the sequencing, RT-qPCR assay, validation results, seekCRIT output format, supplementary tables A, B, and supplementary figures A through F can be found here.
(DOCX)

S1 Table Differentially expressed circular RNAs. Differentially expressed circular RNAs (DECs) detected from seekCRIT analyses on three datasets: Somata vs. Neuropil, Normaxia vs. Hypoxia, and CTRL vs. IR12.
(XLSX)

S1 File seekCRIT package with test data. Source code, license, usage, and test data can be found here.
(ZIP)
