# Supplementary material for: seekCRIT: Detecting and characterizing differentially expressed circular RNAs using high-throughput sequencing data
Source: PLoS Comput Biol. 2020 Oct 20;16(10):e1008338. doi: 10.1371/journal.pcbi.1008338 (PMC7598922; doi:10.1371/journal.pcbi.1008338)
Supplement: S1 Text — The details of the sequencing, RT-qPCR assay, validation results, seekCRIT output format, supplementary tables A, B, and supplementary figures A through F can be found here. (DOCX) [file pcbi.1008338.s001.docx]

seekCRIT: detecting and characterizing differentially expressed circular RNAs using high-throughput sequencing data

Mohamed Chaabane^1¶&^, Kalina Andreeva^2¶^, Jae Yeon Hwang^1^, Tae Lim Kook^1^, Juw Won Park^1,3*^, Nigel G. F. Cooper^2,3*^

^1^Department of Computer Science and Engineering, ^2^Department of Anatomical Sciences and Neurobiology, ^3^KBRIN Bioinformatics Core, University of Louisville, Louisville, KY 40292, USA

* Corresponding authors

^¶^ These authors contributed equally to this work.

^&^ current address: Department of Computer Science, Colorado State University, Fort Collins, CO 80526, USA

**RNA-Seq**

RNA was extracted using the RNeasy Lipid Tissue Mini Kit (Qiagen). Sequencing libraries were prepared using the TrueSeq Stranded Total RNA LT Sample Prep Kit (Illumina) with Ribo-Zero Gold according to the manufacturer's instructions. 350ng of samples (in a volume of 10 μl) were treated with Ribo-Zero rRNA binding Buffer, rRNA removal Solution and RNase free water to the final volume of 20 μl and denatured for 5 minutes at 68⁰C. Then rRNA was removed by adding the probe hybridized RNA sample to the rRNA removal beads. The RNA quality and quantity were evaluated using Nanodrop 2000 (Thermo Scientific) and Bioanalyzer (Agilent 2100). The samples were sequenced on a NextSeq 550 System using 4 lines and single-end protocol to generate 150 bp reads.

**RT-qPCR assay**

RT-qPCR assays were performed in ABI PRISM 7900HT Sequence Detection System (Applied Biosystems, Foster City, CA) employing SYBR green detection. 1μg RNA in 20µl reaction and random hexamer primers were used for the synthesis of cDNA, by application of High-Capacity cDNA Reverse Transcription Kit (Applied Biosystems Foster City, CA) and following the manufacturer’s instructions. cDNA was diluted (1:13) and one microliter of the dilution was used in each reaction along with 10 μL SYBR Green PCR Master Mix (Cat# 4309155, Applied Biosystems, Foster City, CA), 1 μL from each of the forward and reverse exon-exon-specific custom designed primers (5μM each) and 7 μL deionized water in a final reaction volume of 20 μL. Each qPCR reaction was done in 3 technical replicates and all samples were run on a single 384 well plate. GAPDH was used as reference (internal control). The primers for the target and reference genes were generated by IDT (Coralville, IA, USA) and their sequences are listed in **Table A**. **Fig A** illustrates the positioning of the primers for amplification of the exon-exon linear and circular junctions. qPCR Ct values were calculated automatically using the manufacturer’s software (SDS v2.3 RevA, Applied Biosystems, Foster City, CA). Expression of targeted exon junctions was calculated with the ΔΔCTmethod (in which ΔCT = CT of the target gene junction – CT of GAPDH, and calibrator = mean ΔCT of all junctions of the target gene). For each exon-exon junction, the relative level of gene expression was then converted into fold-difference relative to the calibrator as 2−ΔΔCT as described by Livak and Schmittgen (58, 59).

**Rat retina data RT-qPCR validation results**

The result for one randomly chosen circRNA from each category (upregulated, downregulated and unchanged) is shown in **Fig D**. circRims2 was predicted and confirmed as upregulated in IR-injured retina compared to the sham control. Its upregulation is supported by the higher number of reads in the IR-sample (93 reads) when compared to the number of reads in the sham control (17 reads). Similarly, higher fold change of circRims2 expression was detected in the IR12-sample (0.34) than in the CTRL (0.23). These values, however, are not very informative unless they are compared to the abundance of the surrounding linear junctions. A good example for demonstrating this point is the circRNA Bbs5 that is downregulated in response to the ischemic insult. The number of reads for the circular back splice junctions of circBbs5 do not show a large change when comparing IR12 to CTRL (58 vs 60 reads, respectively). In agreement with the numbers of reads, the difference in fold change in the expression of circBbs5 appears to be very low (0.66 in IR12 vs. 0.71 in CTRL). However, relative to the abundance of the linear junctions in both conditions (IR12 and CTRL), circBbs5 is downregulated in retinal ischemia by 8% based on the deltaPBI_RT-qPCR and 23% based on the deltaPBI_seekCRIT (**Fig D**).

The results described above indicate that the RT-qPCR and the NGS approaches have different levels of sensitivity/efficacy in estimating the abundance of different exon junctions within the same gene and in different genes. As a result of this difference, we performed a Pearson’s correlation which indicated agreement (r = 0.71) between the trends determined by seekCRIT and RT-qPCR (see **Fig C**). The failed validation was for circRNA circRiok, and we noted that this circRNA had the lowest number of circular junction reads (10 reads in the IR sample vs 2 reads in the sham control sample, see **Table B**). We are not certain that RT-qPCR based on SYBR chemistry is sensitive enough to detect a difference of 8 reads (or to detect the exact amount of fluorescence produced from 2 reads only). In addition, the 5 control circRNAs (for which seekCRIT detected no change) did not show clear directional change when comparing CTRL to IR12. One (*Sclt1*) of the 5 circRNAs was upregulated while two (*Gucy1a2* and *Phkb*) were downregulated. The other two (*Ash1l* and *Pclo*) did not change more than 5% (**Table B**). Based on these results, the RT-qPCR data confirm the validity of the tool, and together, these findings indicate that some circRNAs are implicated in the etiology of ischemia-reperfusion injury in the retina.

**seekCRIT output**

The output of seekCRIT contains 21 columns describing differentially expressed circular RNAs. It is an extension of bed file format.

| **Field** | **Description** |
| --- | --- |
| chrom | chromosome |
| circRNA_start | circular RNA 5' end position |
| circRNA_end | circular RNA 3' end position |
| strand | DNA strand (+/-) |
| exonCount | number of exons included in the circular RNA transcript |
| exonSizes | size of exons included in the circular RNA transcript |
| exonOffsets | offsets of exons included in the circular RNA transcript |
| circType | circRNA, ciRNA, ccRNA |
| geneName | name of gene |
| isoformName | name of isoform |
| exonIndexOrIntronIndex | Index (start from 1) of exon (for circRNA) or intron (for ciRNA) in given isoform |
| FlankingIntrons | Left intron/Right intron |
| CircularJunctionCount_Sample_1 | read count of the circular junction in sample # 1 |
| LinearJunctionCount_Sample_1 | read count of the linear junction in sample # 1 |
| CircularJunctionCount_Sample_2 | read count of the circular junction in sample # 2 |
| LinearJunctionCount_Sample_2 | read count of the linear junction in sample # 2 |
| PBI_Sample_1 | Percent Backspliced In for sample # 1 |
| PBI_Sample_2 | Percent Backspliced In for sample # 2 |
| deltaPBI(PBI_1-PBI_2) | difference between PBI values of two samples |
| pValue | pValue |
| FDR | False discovery rate |

| **Table A.** Sequences of the primers used to determine the relative accumulation of back-spliced exon junctions and their surrounding linear exon junctions. | |
| --- | --- |
| **Primer name** | **Primer sequence** |
| Rims2_E23_f | GTAGTGCTTCTCGTTTCAGC |
| Rims2_E24_r | CTCCAGTGAGCACATGTCTC |
| Rims2_E25_f | ACTGTCCAGAGAAGCACGGA |
| Rims2_E25_f1 | AGGAACTGGATGACAAGACAGGC |
| Rims2_E26_r1 | TCTGGCGTCCCACTAGCTGA |
| Fat3_E2_f | ATGGAGAGATCCAGTACTCC |
| Fat3_E2_r | TGCGTGAAGTGGAAGCCCAA |
| Fat3_E3_r | GTAGACCTCAATGGTGGAGT |
| Camsap1_E1_f | GGATCTGTGCGAAGGCCTAT |
| Camsap1_E2_r | ACACGGCAGTACAGCTCACT |
| Camsap1_E3_f | GTGGCCAGTGTCAAGCGTTT |
| Camsap1_E4_r | TGATGAGCGGGACTTTCCAG |
| Gucy2f_E2_f | TGGGTGCATACAGCCAATCA |
| Gucy2f_E3_r | GACGTAAGTCCCATCCGTCA |
| Gucy2f_E8_f | TCCTGGAGATTTTGGGGACA |
| Gucy2f_E9_r | CTTGATGAGGTCAAGCAGCA |
| Atxn1_E1_r | ATGGCAGACAGATTCGTAGC |
| Atxn1_E3_f | GTAGCTGCTTGTGGCTTTG |
| Atxn1_E4_r | CTCTGACGACAGCAGGTTTG |
| Smad4_Ex1_r | GCGTCATTGCTTGTCGGT |
| Smad4_Ex4_f | TCAGAGTCTAATGCCACC |
| Smad4_Ex5_r | AGTAGCTGGCTGAGCAGTGA |
| Suclg2_E2_f | ACCAGAGCAAGAAGCTCATG |
| Suclg2_E3_r | AAACCGCTATCGAAGACACC |
| Suclg2_E9_f | AGGTGGTGTAAAGGAGTCCC |
| Suclg2_E10_r | TTGGTGATTCCGTTGGCAATGA |
| Strn3_E1_f | CGGGAATACTGCACTACATC |
| Strn3_E2_r | CTTGACCTTTCCTTTCACCTTG |
| Strn3_E19_f | CTGCCCATGAAGATAGACAC |
| Strn3_E20_r | CCATTGGGATCTACTGCT |
| Bbs5_E1_f | GTCTGTGCTGGACGTGTT |
| Bbs5_E2_r | ATAAGGACTTCCCCAGGT |
| Bbs5_E7_f | GTGAGAATTGTGTGGCATG |
| Bbs5_E8_r | GCTGAGAGCTTTCTATGAC |
| Riok1_E5_f | TCGCATTAAAGACAAGGCCG |
| Riok1_E6_r | GATGCAGCCATGTATCTCTG |
| Riok1_E8_f | CGTGGCTATTGCAAAGGAAAC |
| Riok1_E8_f1 | TGGTGAAGACGTGGGCAGA |
| Riok1_E9_r | GGAGAACATGACTTCTTAGCC |
| Gucy1a2_E3_f | GATGCTCCTCTGCAGACCAT |
| Gucy1a2_E4_r | ATGTGCTGCCTACAGCTCGA |
| Gucy1a2_E4_f | ACTTGGAGAGGGCCTACGAAA |
| Gucy1a2_E5_r | TGTCTGAGAGATGCAGTCCT |
| Ash1l_E3_f | TAACTCGAGCTGCCGGAAGAA |
| Ash1l_E4_r | ACCTTTTACGGGTAGGGCTA |
| Ash1l_E5_f | ATGTGATCGAGGCTGTGGTT |
| Ash1l_E6_r | TTCTCGTGGGATGAGAGCCA |
| Sclt1_E5_f | GGGTGAGATGAAACTACAACTTG |
| Sclt1_E5_f1 | CTTGAAACCGTCATCACAGA |
| Sclt1_E6_r | ACGGTCTCATCATCTGCACA |
| Sclt1_E18_f | AGCTCAACAAAAGACGGCCA |
| Sclt1_E19_r | CAGCTACCTTCTCATTTGCC |
| Phkb_E1_f | AGTAAGCTGGAAGCTCTTGG |
| Phkb_E2_r | CATTATCAGGTCTTGGAAG |
| Phkb_E7_f | GTTTACCGTGTTCCCGACT |
| Phkb_E8_r | CTTCTAAGGCTGCTTTTGCC |
| Pclo_E3_f | CAACACCACTGAACTTCTG |
| Pclo_E4_r | TGTGGTGAGGATGGGATTGC |
| Pclo_E8_f | CAACTTCAACACCTGCACAG |
| Pclo_E9_r | GGTGGCATTTTGCCCATATC |
| GAPDH_f | GAGAAACCTGCCAAGTATG |
| GAPDH_r | CTCCTTGGAGGCCATGTAG |

**Table B.** Validation of circular RNAs detected from seekCRIT

|  |  | **seekCRIT** | | | | | | | **qPCR** | | |  |
| --- | --- | --- | --- | --- | --- | --- | --- | --- | --- | --- | --- | --- |
| **Category** | **gene symbol** | **CJC**  **CTRL** | **LJC**  **CTRL** | **CJC**  **IR12** | **LJC**  **IR12** | **PBI**  **CTRL** | **PBI**  **IR12** | **Delta**  **PBI** | **PBI**  **CTRL** | **PBI**  **IR12** | **Delta**  **PBI** | **Validated** |
| Upregulated | Rims2 | 17 | 82 | 93 | 122 | 0.29 | 0.60 | **-0.31** | 0.28 | 0.34 | **-0.059** | **yes** |
|  | Fat3 | 5 | 65 | 29 | 84 | 0.13 | 0.40 | **-0.27** | 0.11 | 0.12 | **-0.002** | **yes** |
|  | Camsap1 | 29 | 343 | 94 | 358 | 0.14 | 0.34 | **-0.19** | 0.01 | 0.04 | **-0.023** | **yes** |
|  | Gucy2f | 19 | 601 | 49 | 554 | 0.05 | 0.15 | **-0.09** | 0.09 | 0.10 | **-0.016** | **yes** |
|  | Atxn1 | 55 | 495 | 102 | 478 | 0.18 | 0.29 | **-0.11** | 0.51 | 0.61 | **-0.093** | **yes** |
|  |  |  |  |  |  |  |  |  |  |  |  |  |
| Downregulated | Smad4 | 56 | 40 | 89 | 237 | 0.73 | 0.42 | **0.30** | 0.15 | 0.13 | **0.024** | **yes** |
|  | Suclg2 | 20 | 62 | 5 | 111 | 0.39 | 0.08 | **0.30** | 0.14 | 0.07 | **0.075** | **yes** |
|  | Strn3 | 29 | 110 | 25 | 237 | 0.34 | 0.17 | **0.17** | 0.12 | 0.08 | **0.048** | **yes** |
|  | Bbs5 | 60 | 136 | 58 | 385 | 0.46 | 0.23 | **0.23** | 0.31 | 0.23 | **0.081** | **yes** |
|  | Riok1 | 10 | 78 | 2 | 188 | 0.20 | 0.02 | **0.18** | 0.05 | 0.078 | **-0.026** | **no** |
|  |  |  |  |  |  |  |  |  |  |  |  |  |
| Unchanged | Gucy1a2 | 83 | 79 | 77 | 73 | 0.67 | 0.67 | **-0.01** | 0.33 | 0.39 | **-0.056** | **no** |
|  | Ash1l | 124 | 405 | 305 | 999 | 0.37 | 0.37 | **0.001** | 0.25 | 0.20 | **0.048** | **yes** |
|  | Sclt1 | 55 | 79 | 77 | 113 | 0.58 | 0.57 | **0.005** | 0.66 | 0.60 | **0.054** | **no** |
|  | Phkb | 50 | 54 | 46 | 52 | 0.64 | 0.63 | **0.010** | 0.54 | 0.64 | **-0.102** | **no** |
|  | Pclo | 13 | 84 | 18 | 117 | 0.23 | 0.23 | **0.001** | 0.27 | 0.30 | **-0.032** | **yes** |


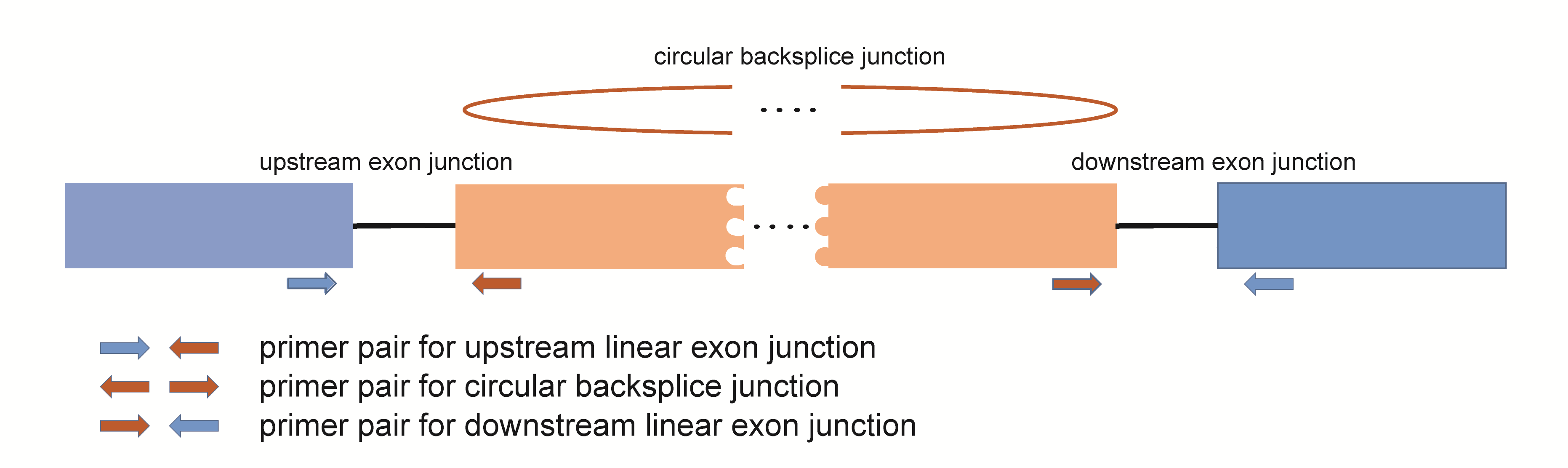


**Fig A** Positioning of the primers for amplification of the exon-exon linear and circular junctions.


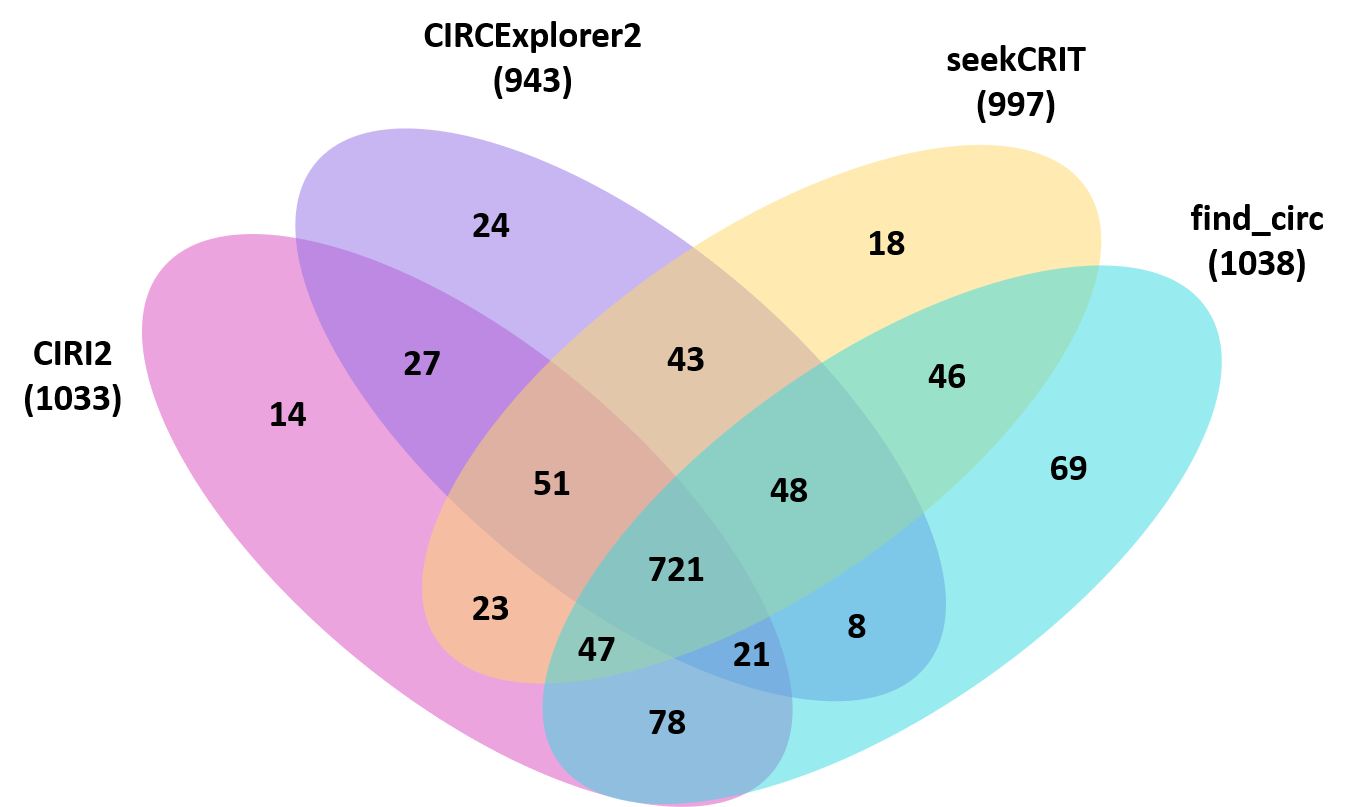


**Fig B** Venn Diagram of detected circular RNAs from different tools. Rat brain tissue (somata layers of CA1 hippocampal region, no replicates, GSE61991). This comparison showed that majority of detected circRNAs overlap among tools.


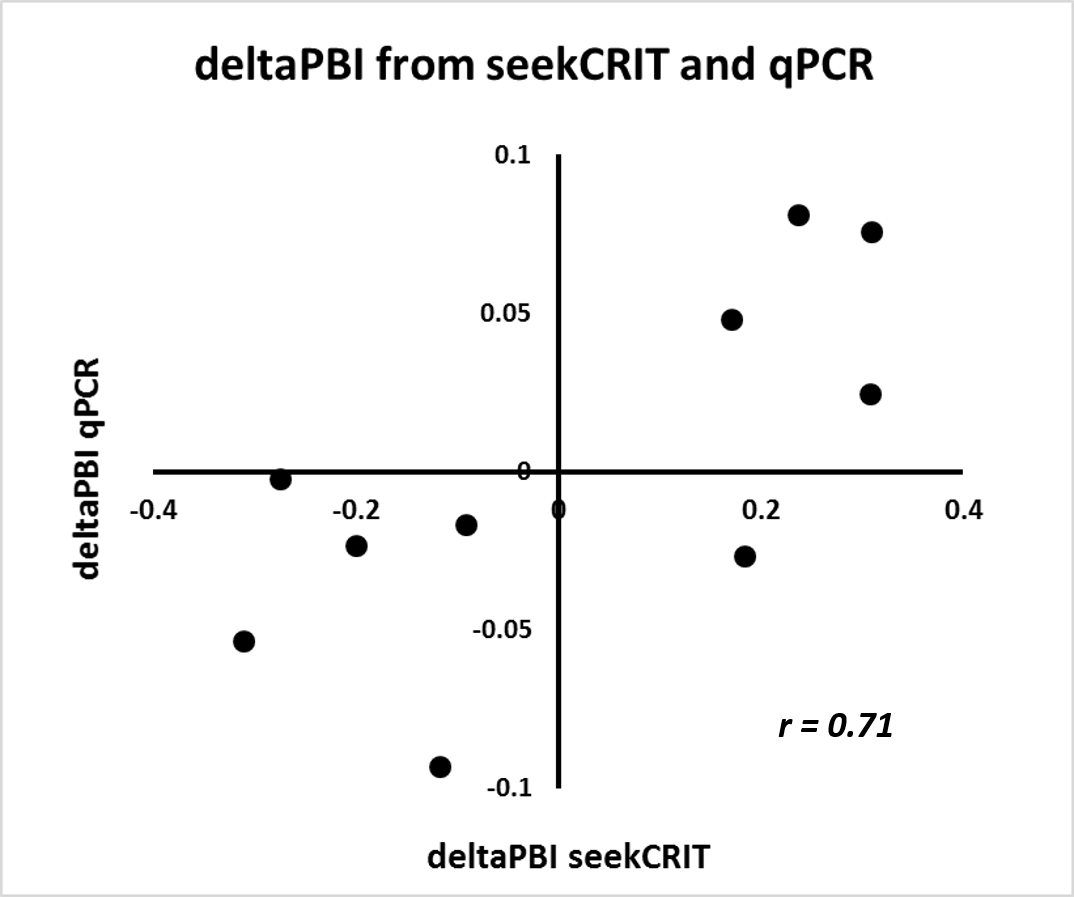


**Fig C** deltaPBI estimates from seekCRIT (x-axis) and qPCR (y-axis) showed high correlation (Pearson’s *r* >0.7) for 10 randomly selected DECs.


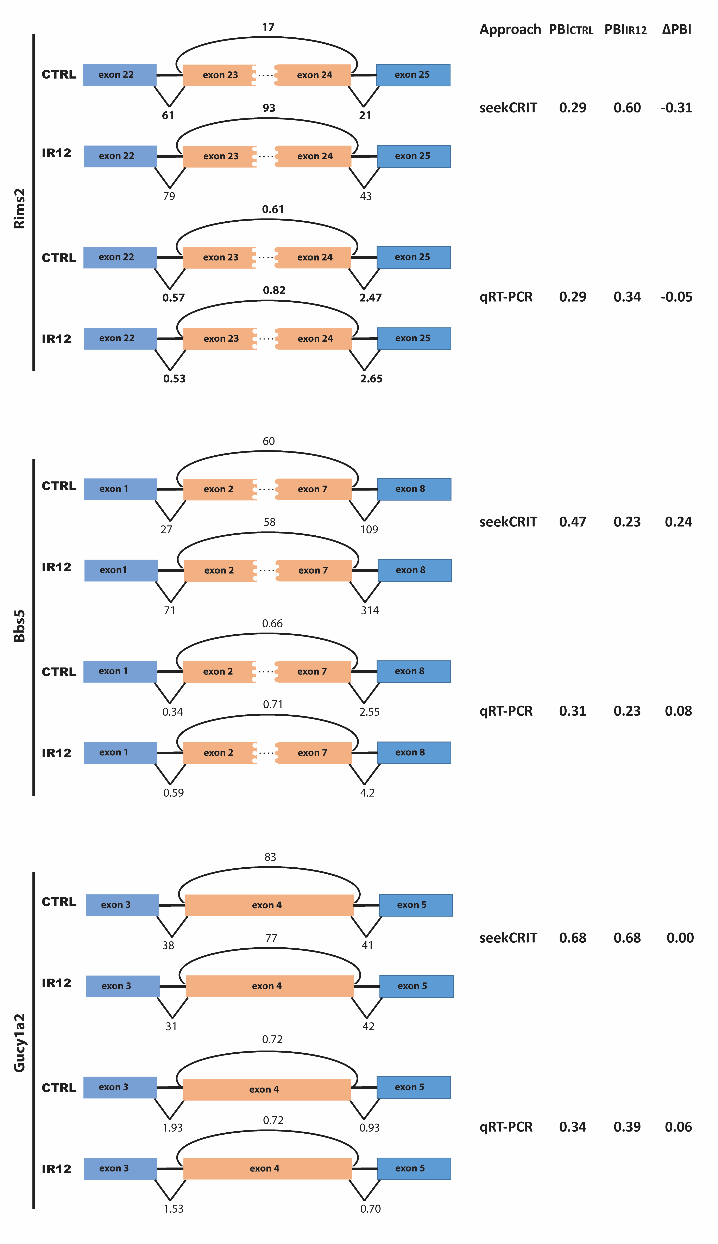


**Fig D** RNA-seq and RT-qPCR analysis of circRNAs. One circRNA per each category is shown. From the top, upregulated (*Rims2*), downregulated (*Bbs5*), and unchanged (*Gucy1a2*).


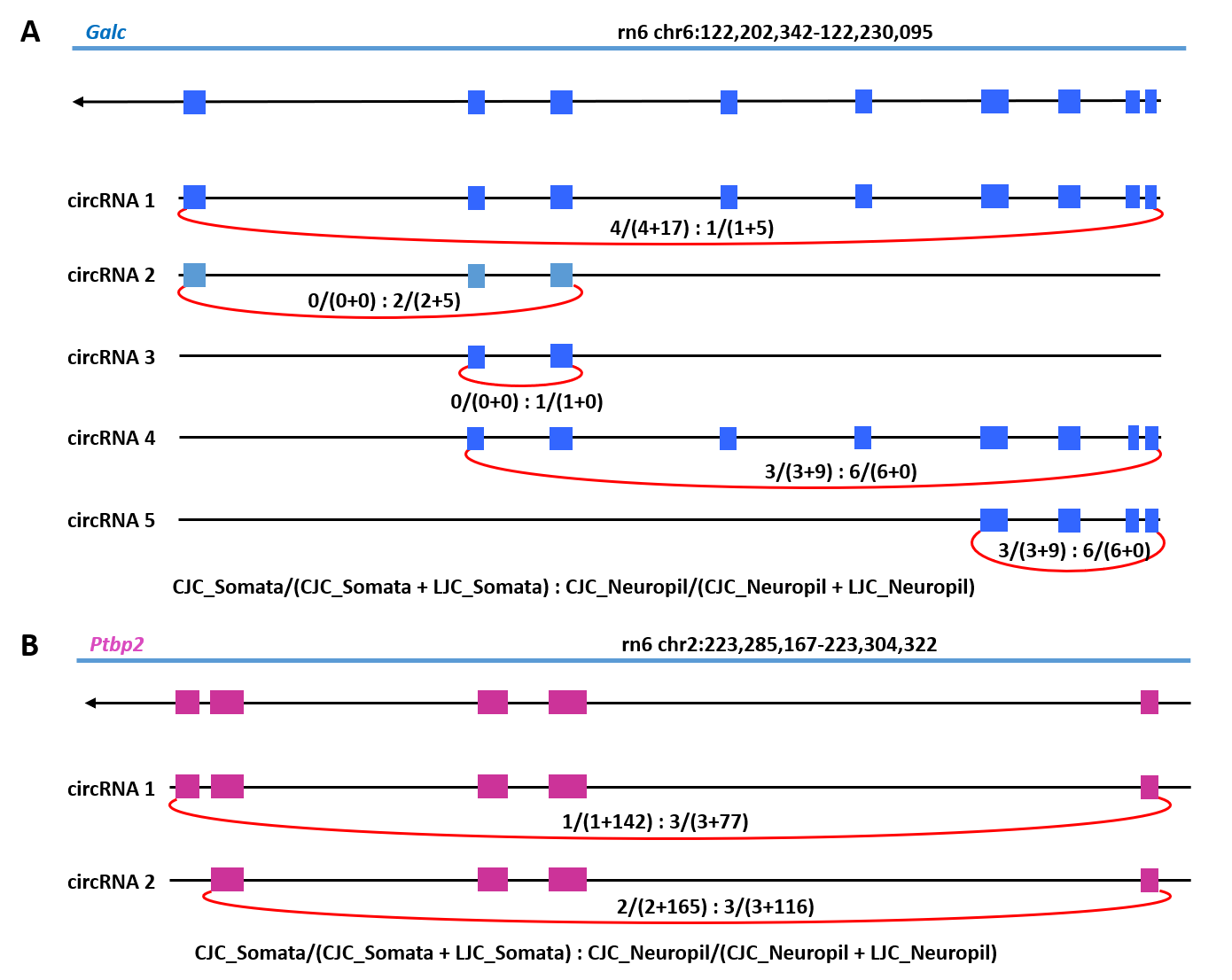


**Fig E** Multiple circular RNAs can be derived from one gene. seekCRIT detected this phenomenon from rat brain tissue data (somata and neuropil layers of CA1 hippocampal region, no replicates, GSE61991). **(A)** Five circular RNAs derived from Galc. **(B)** Two circular RNAs derived from Ptbp2. Circular formation, circular junction count (CJC) and linear junction count (LJC) are shown.

**
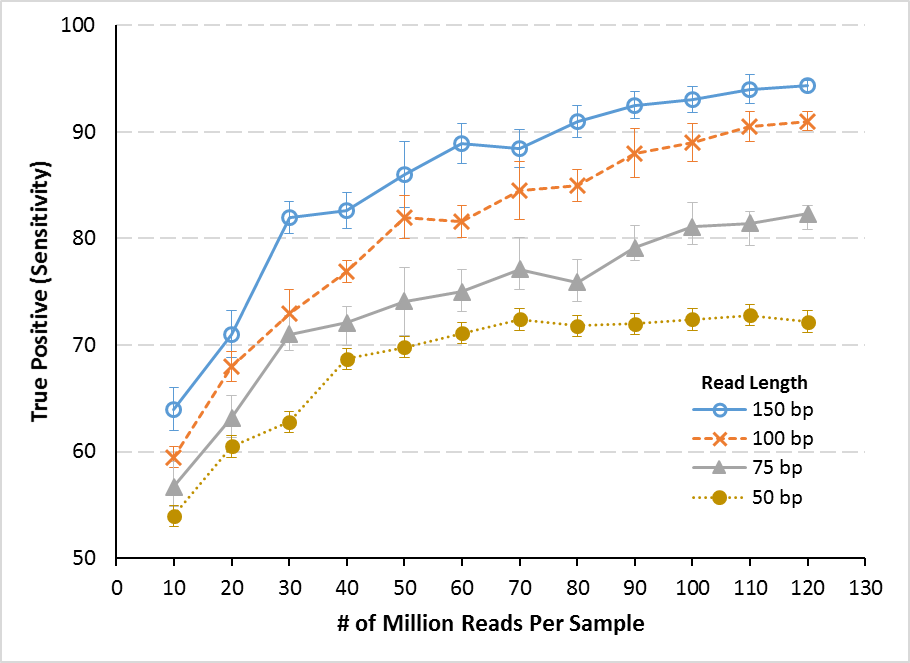
**

**Fig F** Simulation study of seekCRIT with various levels of read length and sequencing depth. Longer reads and deeper sequencing provide higher sensitivity. The error bar indicates the standard error of the mean (SEM) from 10 independent random samplings at each sequencing depth (from 10M up to 120M per sample) and at each read length (50-150 bp).
