# Supplementary material for: seekCRIT: Detecting and characterizing differentially expressed circular RNAs using high-throughput sequencing data
Source: PLoS Comput Biol. 2020 Oct 20;16(10):e1008338. doi: 10.1371/journal.pcbi.1008338 (PMC7598922; doi:10.1371/journal.pcbi.1008338)
Supplement: S1 File — Source code, license, usage, and test data can be found here. (ZIP) [file pcbi.1008338.s003.zip › Instruction_seekCRIT.pdf]

# seekCRIT

seek for Circular RNA In Transcriptome (identifies deferentially expressed circRNAs between two samples)

Version: v1.0.0.b

Last Modified: 2019-06-25

Authors: Bioinformatics Lab (http://bioinformatics.louisville.edu/lab/index.php), University of Louisville, Kentucky Biomedical Research Infrastructure Network (KBRIN) (http://louisville.edu/research/kbrin/)

## Prerequisites

### Software / Package

- STAR Aligner (https://github.com/alexdobin/STAR): v2.5.2b or later

### Others

- pysam (https://github.com/pysam-developers/pysam): v0.9.1.4 or later
- numpy (https://github.com/numpy/numpy): v1.11.2 or later
- scipy (https://docs.scipy.org/doc/scipy/reference/dev/index.html)
- fisher (Fisher's exact test) (https://pypi.python.org/pypi/fisher/)
- mne (FDR calculation) (https://github.com/mne-tools/mne-python/tree/master/mne)

## Installation

1. Download "seekCRIT-master.zip" file

2. Unzip the file

3. Install required packages (Some prerequisite packages might require admin access rights. Please contact your system admin to install such packages.)

```
pip3 install -r Prerequisites.txt
```

4. Install seekCRIT

```
python3 setup.py install
```

5. testing seekCRIT with testrun.sh

In order to run testrun.sh:

- Dowload genome sequence in FASTA format for *Rattus norvegicus* genome (ftp://hgdownload.soe.ucsc.edu/goldenPath/rn6/bigZips/rn6.fa.gz). Unzip the file and save as "seekCRIT/fasta/rn6.fa"

- Dowload gtf annotation for *Rattus norvegicus* genome (ftp://ftp.ensembl.org/pub/release-84/gtf/rattus_norvegicus/Rattus_norvegicus.Rnor_6.0.84.gtf.gz). Unzip the file and save as "seekCRIT/gtf/Rattus_norvegicus.Ensembl.rn6.r84.gtf"

- Run testrun.sh to test the installation and the dependencies of seekCRIT. It will also test the installation with files, "CRTL12.fastq" and "IR12.fastq" in the folder "testData" by specifying the path for FASTA and gtf files:

```
./testrun.sh gtf/Rattus_norvegicus.Ensembl.rn6.r84.gtf fasta/rn6.fa
```

## Usage

```
usage: seekCRIT.py [-h] -s1 S1 -s2 S2 -gtf GTF -o OUTDIR -t {SE,PE}
               --genomeIndex GENOMEINDEX -fa FASTA -ref REFSEQ
```

```
               [--threadNumber numThreads]
               [--aligner aligner]
               [--deltaPSI DELTAPSI] [--highConfidence HIGHCONFIDENCE]
               [--libType {fr-unstranded,fr-firststrand,fr-secondstrand}]
               [--keepTemp {Y,N}]

Identifying and Characterizing Differentially Spliced circular RNAs between two
samples

Required arguments:
===================
  -s1 S1, --sample1 S1  fastq files for sample_1. Replicates are separated by
                        comma. Paired-end reads are separated by colon.
                        e.g.,s1-1.fastq,s1-2.fastq for single-end read. s1-1.R
                        1.fastq:s1-1.R2.fastq,s1-2.R1.fastq:s1-2.R2.fastq for
                        single-end read

  -s2 S2, --sample2 S2  fastq files for sample_2. Replicates are separated by
                        comma. Paired-end reads are separated by colon.
                        e.g.,s2-1.fastq,s2-2.fastq for single-end read. s2-1.R
                        1.fastq:s2-1.R2.fastq,s2-2.R1.fastq:s2-2.R2.fastq for
                        single-end read

  -gtf GTF, --gtf GTF   The gtf annotation file. e.g., hg38.gtf

  -o OUTDIR, --output OUTDIR
                        Output directory

  -t {SE,PE}, --readType {SE,PE}
                        Read type. SE for Single-end read, PE for Paired-end read

  --genomeIndex GENOMEINDEX
                        Genome indexes for the aligner

  -fa FASTA, --fasta FASTA
                        Genome sequence. e.g., hg38.fa

  -ref REFSEQ, --refseq REFSEQ
                        Transcriptome in refseq format. e.g., hg38.ref.txt

 optional arguments:
====================

   -h, --help            show this help message and exit
   --threadNumber numberOfThreadsk
                        Number of threads for multi-threading feature [default =
                        4]
  --aligner aligner     aligner to use(for now it supports only STAR but we are
                        working on it to support more aligners)

  --deltaPSI DELTAPSI   Delta PSI cutoff. i.e., significant event must show
                        bigger deltaPSI than this cutoff [default = 0.05]

  --highConfidence HIGHCONFIDENCE
                        Minimum number of circular junction counts required
```

```
                            [default = 1]

        --libType  {fr-unstranded,fr-firststrand,fr-secondstrand}
                            library type used by Tophat aligner [default ='fr-
                            unstranded']

        --keepTemp {Y,N}      Keep temp files or not  [default='Y']
```

## Example

### Paired-end reads

```
python3 seekCRIT.py -o PEtest -t PE -fa fa/hg19.fa -ref ref/hg19.ref.txt --
genomeIndex /media/bio/data/STARIndex/hg19 -s1 testData/231ESRP.25K.rep-
1.R1.fastq:testData/231ESRP.25K.rep-1.R2.fastq,testData/231ESRP.25K.rep-
2.R1.fastq:testData/231ESRP.25K.rep-2.R2.fastq -s2 testData/231EV.25K.rep-
1.R1.fastq:testData/231EV.25K.rep-1.R2.fastq,testData/231EV.25K.rep-
2.R1.fastq:testData/231EV.25K.rep-2.R2.fastq -gtf testData/test.gtf --threadNumber
12
```

### Single-end reads

```
python3 seekCRIT.py -o SEtest -t SE -fa fa/hg19.fa -ref ref/hg19.ref.txt --
genomeIndex /media/bio/data/STARIndex/hg19 -s1 testData/231ESRP.25K.rep-
1.R1.fastq,testData/231ESRP.25K.rep-1.R2.fastq,testData/231ESRP.25K.rep-
2.R1.fastq,testData/231ESRP.25K.rep-2.R2.fastq -s2 testData/231EV.25K.rep-
1.R1.fastq,testData/231EV.25K.rep-1.R2.fastq,testData/231EV.25K.rep-
2.R1.fastq,testData/231EV.25K.rep-2.R2.fastq -gtf testData/test.gtf --threadNumber
12
```

## Note

- Transcriptome should be in refseq format below (see more details in the example file "hg19.ref.txt" (https://github.com/UofLBioinformatics/seekCRIT/blob/master/example/hg19.ref.txt)):

| Field | Description |
|---|---|
| geneName | Name of gene |
| isoform_name | name of isoform |
| chrom | chromosme |
| strand | strand (+/-) |
| txStart | Transcription start position |
| txEnd | Transcription end position |
| cdsStart | Coding region end |
| exonCount | Number of exons |
| exonStarts | Exon start positions |
| exonEnds | Exon end positions |

- It is not obligatory to provide REFSEQ file, we made script (GTFtoREFSEQ) to convert from gtf to refseq that is used in the main code if no refseq file is provided.

**Output**

See details in the example file, “circRNAs.pVal.FDR.txt”
(https://github.com/UofLBioinformatics/seekCRIT/blob/master/example/circRNAs.pVal.FDR.txt)

| Field | Description |
|---|---|
| chrom | Chromosome |
| circRNA_start | circular RNA 5' end position |
| circRNA_end | circular RNA 3' end position |
| strand | DNA strand (+/-) |
| exonCount | number of exons included in the circular RNA transcript |
| exonSizes | size of exons included in the circular RNA transcript |
| exonOffsets | offsets of exons included in the circular RNA transcript |
| circType | circRNA, ciRNA, ccRNA |
| geneName | name of gene |
| isoformName | name of isoform |
| exonIndexOrIntronIndex | Index (start from 1) of exon (for circRNA) or intron (for ciRNA) in given isoform |
| FlankingIntrons | Left intron/Right intron |
| CircularJunctionCount_Sample_1 | read count of the circular junction in sample # 1 |
| LinearJunctionCount_Sample_1 | read count of the linear junction in sample # 1 |
| CircularJunctionCount_Sample_2 | read count of the circular junction in sample # 2 |
| LinearJunctionCount_Sample_2 | read count of the linear junction in sample # 1 |
| PBI_Sample_1 | Percent Backsplicing Index for sample # 1 |
| PBI_Sample_2 | Percent Backsplicing Index for sample # 2 |
| deltaPBI(PBI_1-PBI_2) | difference between PBI values of two samples |
| pValue | pValue |
| FDR | FDR |

To calculate the significane of differentially expressed circular RNAs, use the criteria:
- At least 5% changes in percent back-spliced in (PBI) or |deltaPBI|>=5%
- FDR < 0.05

**License**

Copyright (C) 2017.  See the LICENSE file for license rights and limitations (MIT)
(https://github.com/UofLBioinformatics/see-kCRIT/blob/master/LICENSE).
